# A Follow-Up Report of an Infant Born to a Mother Receiving Tamoxifen

**DOI:** 10.1155/2020/2056756

**Published:** 2020-07-26

**Authors:** Yuki Okada, Daisuke Hitaka, Takahiro Kido, Yu Kanai, Kumi Konishi, Kosuke Doki, Nobuko Katayama, Masato Homma, Yayoi Miyazono, Hidetoshi Takada

**Affiliations:** ^1^Department of Pediatrics, University of Tsukuba Hospital, Tsukuba, Ibaraki, Japan; ^2^Department of Pharmacy, University of Tsukuba Hospital, Tsukuba, Ibaraki, Japan; ^3^Department of Child Health, Seinan Medical Center Hospital, Sakai, Ibaraki, Japan; ^4^Department of Pharmaceutical Sciences, Faculty of Medicine, University of Tsukuba, Tsukuba, Ibaraki, Japan; ^5^Department of Child Health, Faculty of Medicine, University of Tsukuba, Tsukuba, Ibaraki, Japan

## Abstract

Tamoxifen, an estrogen receptor antagonist, is contraindicated in pregnant women due to its teratogenic activity. In the present study, we report the case of an infant whose mother received tamoxifen for breast cancer while unaware of the pregnancy. The infant, born at 29 weeks and 6 days of gestational age with a birth weight of 1664 g, had no congenital anomalies. This case presents detailed information on the development of an infant with placental transfer of tamoxifen. The infant has grown and developed normally throughout a 5-year follow-up period, but long-term vigilance continues.

## 1. Introduction

Pregnancy complicated by malignancy is relatively common, with a frequency of approximately 1 in 1,000 [1]. Breast cancer is the most frequently diagnosed malignancy during pregnancy, occurring approximately once in 3,000 pregnancies [2]. However, tamoxifen, a first line therapy for breast cancer, is contraindicated for administration during pregnancy owing to its risk for teratogenicity [3]. In the present study, we report an infant whose mother received tamoxifen for breast cancer while unaware of the pregnancy. This is the first follow-up report on an infant who was exposed to tamoxifen during the fetal period, with a definite level of tamoxifen and its metabolites seen in the blood just after delivery [4].

## 2. Case Presentation

**Figure 1:**
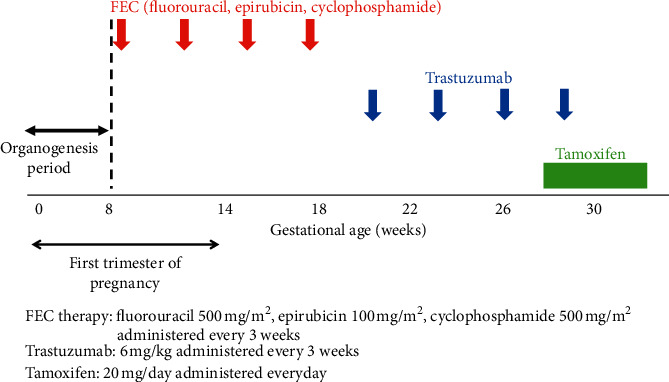
Time course of the administration of anticancer agents.

A 39-year-old woman underwent a total mastectomy for breast cancer (clinical stage IIIA) and, thereafter, waited 1 month before completing four courses of treatment with fluorouracil, epirubicin, and cyclophosphamide (FEC therapy). Thereafter, she received four courses of trastuzumab and tamoxifen for roughly 7 consecutive weeks until the day before delivery (Figure 1). At the initiation of chemotherapy, she experienced amenorrhea and lower abdominal distension. However, a commercially available pregnancy test was negative, which might have been a false-negative result owing to high amounts of hCG in the urine, the so-called “hook effect”; the symptoms were, therefore, considered as treatment side effects. Thus, ultrasound testing for pregnancy was not conducted and discontinuation of tamoxifen was never considered. As a result, the mother had been receiving tamoxifen until the delivery. Five months after the initial administration of chemotherapy, she experienced a strong lower abdominal pain without fever and delivered a baby at an emergency outpatient unit. This was the first time that her pregnancy was noticed.

**Figure 2:**
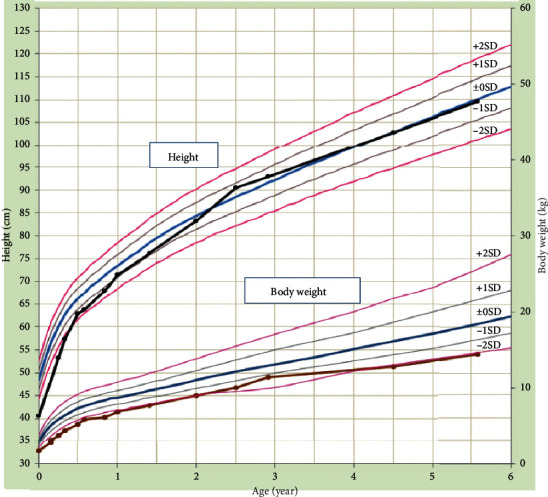
Growth curve of the patient's daughter.

The female neonate had a birth weight of 1664 g, a height of 40.5 cm, and a head circumference of 30 cm with Apgar scores of unknown (1 min) and 7 (5 min). Owing to a respiratory disorder, the infant was intubated and transported to our hospital. The gestational age as estimated from the last menstrual period remembered by the mother was 29 weeks and 6 days; however, the Dubowitz neurological assessment placed the gestational age at approximately 32–34 weeks. The infant was diagnosed with respiratory distress syndrome, received surfactant replacement therapy, and gradually improved until extubation on day 3 of life. The infant had both good feeding and weight gain with no findings of malformation on echocardiography, abdominal ultrasonography (performed at day 0), or brain magnetic resonance imaging (performed at day 39). The infant was discharged at 1 month of age. Nearly normal growth and motor and mental development have been continuously observed within the infant over 5 years of follow-ups with the interval of every 3–6 months. Her height has been over −2 standard deviation (SD) for Japanese children since the age of 6 months, and her body weight has been over −2 SD since 2 years of age. At 5 years of age, her height was 109.5 cm (−0.1 SD) and weight was 14.4 kg (−2.0 SD) (Figure 2). She could hold her head steady at 4 months of age. She could not perform thumb-finger grasping until she was 1 year old. She could walk alone at 1 year and 5 months, and she could run and speak 2-word sentences at 2 years of age. Her intelligence was deemed to be within the normal range, as the result obtained after evaluation using the Tanaka–Binet intelligence scale was equivalent to an IQ of 94 when she was 4 years old.

Blood concentrations of tamoxifen and its metabolites (N-desmethyl tamoxifen, 4-hydroxy tamoxifen, endoxifen) were determined in the mother and the infant at 3-4 hours after birth. The details of drug concentration have been reported before [4]. Similar blood concentrations were observed in mother and infant, confirming mother-to-fetus tamoxifen transmission; to the best of our knowledge, this is the first time this confirmation has been made.

## 3. Discussion

This follow-up report of an infant whose mother received tamoxifen during pregnancy and just before delivery is the first report worldwide in terms of confirming the plasma concentrations of tamoxifen and its metabolites in a neonate. The infant, now 5 years old, has achieved normal development, except for a slight delay in the fine motor activity until 2 years of age. With respect to growth, the infant had been small of built but had caught up in her growth curve by the age of 2 years. Follow-up reports about infants exposed to tamoxifen in utero are scarce; however, Koca et al. described a healthy 2-year-old child exposed to tamoxifen in the fetus for 1.5 months [5]. Ishizuka and Satou described the normal development up to 5 years follow-up of the infant exposed to tamoxifen during the first trimester. The infant was born at term with a birth weight of 2544 g, and tamoxifen administration was ceased at 4 months before birth [6]. Comparing our case with that of Ishizuka and Satou seems to not offer a significant consideration because of differences in the infant's maturity and timing of tamoxifen administration. Although taking tamoxifen during pregnancy is contraindicated by its potential teratogenicity, no obvious anomalies were observed in this study. This may be explained by the administration of tamoxifen after the first trimester.

Regarding development in infants exposed to chemotherapy in utero, prematurity rather than the influence of anticancer agents could be assumed to have a negative impact [7]. Most studies on the long-term development of those infants have not shown a significant association between chemotherapy itself and an infant's postnatal development. In general, the need for an earlier introduction of chemotherapy and worsening of the maternal physical condition are likely to cause preterm deliveries in mothers with cancer [7]. Amant et al. observed that the rate of preterm births (less than 37 weeks) from mothers who received chemotherapy during pregnancy (61.2%) was significantly higher than that in the general population in participating countries (6.8–8%) [8]. Furthermore, in the same study, adverse cognitive outcome correlated with prematurity was documented at median age of 22 months independent of exposure to anticancer agents in utero.

Tamoxifen, a nonsteroidal selective estrogen receptor modulator, provides an estrogen antagonistic effect in breast tissue and an agonistic effect in the uterus and ovaries. The delicate balance between estrogen and progesterone is considered crucial for maintaining pregnancy and the natural induction of labor [9]. In fact, some preterm deliveries during tamoxifen administration in animal studies have been reported [10], although no evidence demonstrates a causal relationship between tamoxifen and preterm labor. On the contrary, based on the potential adverse impact of not taking tamoxifen during pregnancy, Schuurman et al. recommended that in patients with breast cancer who wish to conceive, a choice of either receiving tamoxifen during pregnancy with a disclaimer as to the risks of fetal malformations or discontinuation/postponement of tamoxifen, which may worsen their prognoses can be offered [9]. In this respect, our follow-up report seems to be valuable because of the paucity of reports on human infants exposed to tamoxifen after the first trimester. Certainly, further follow-ups of the infant are warranted, especially regarding genital development, as genital developmental failure and vaginal cancer from puberty have been reported in cases exposed to diethylstilbestrol [11], which has a chemical structure similar to that of tamoxifen, during the fetal period.

## 4. Conclusion

The preterm infant exposed to tamoxifen after first trimester until just before birth has grown and developed normally throughout a 5-year follow-up.
